# Multiomics Integrative Analysis Identifying *EPC1* as a Prognostic Biomarker in Head and Neck Squamous Cell Carcinoma

**DOI:** 10.1155/2022/1074412

**Published:** 2022-09-16

**Authors:** Yongmei Dai, Wenhan Chen, Junpeng Huang, Lijing Zheng, Qing Lin, Tongjian Cui, Chen Huang

**Affiliations:** ^1^Department of Oncology, Shengli Clinical Medical College of Fujian Medical University & Fujian Provincial Hospital, Fujian 350001, China; ^2^The Second Clinical Medical College of Fujian Medical University, Fujian 362000, China; ^3^Department of Clinical Medicine, Fujian Medical University, Fujian 350122, China; ^4^Shengli Clinical Medical College of Fujian Medical University & Fujian Provincial Hospital, Fujian 350001, China

## Abstract

**Background:**

Biomarker research in head and neck squamous cell carcinoma (HNSCC) is constantly revealing promising findings. An enhancer of polycomb homolog 1 (*EPC1*) was found to play a procancer role in nasopharyngeal carcinoma (NPC), but its role in HNSCC with strong heterogeneity is still unclear. Herein, we investigated the prognostic significance and related mechanisms of *EPC1* in HNSCC.

**Methods:**

The Kaplan-Meier plotter was used to evaluate the prognostic significance of *EPC1*. Based on a range of published public databases, the multiomics expression of *EPC1* in HNSCC was explored to investigate the mechanisms affecting prognosis.

**Results:**

According to the clinical data, high *EPC1* expression in HNSCC was a predictor of patient prognosis (hazard ratio (HR) = 0.64; 95% confidence interval (CI) 0.49-0.83; *P* < 0.01). *EPC1* expression varied among clinical subtypes and was related to key factors, such as TP53 and human papillomavirus (HPV) (*P* < 0.05). At the genetic level, *EPC1* expression level may be associated with protein phosphorylation, cell adhesion, cancer-related pathways, etc. For the noncoding region, a competing endogenous RNA network was constructed, and 6 microRNAs and 12 long noncoding RNAs were identified. At the protein level, a protein-protein interaction (PPI) network related to *EPC1* expression was constructed and found to be involved in HPV infection, endocrine resistance, and multiple cancer pathways. At the immune level, *EPC1* expression was correlated with a variety of immune cells and immune molecules, which together constituted the immune microenvironments of tumors.

**Conclusion:**

High *EPC1* expression may predict a better prognosis in HNSCC, as it is more frequently found in HNSCC with HPV infection. *EPC1* may participate in the genomics, transcriptomics, proteomics, and immunomics of HNSCC, and the results can provide a reference for the development of targeted drugs and evaluation of patient prognosis.

## 1. Background

Due to increases in tobacco use and the human papillomavirus (HPV) infection rate, the number of patients with head and neck squamous cell carcinoma (HNSCC) is increasing, which is one of the most common cancers and accounts for approximately 5% of all malignancies [1, 2]. The successful development of targeted therapies in biomarker-selected patients for personalized medicine has shifted expectations in cancer research [3, 4], and the lack of targetable genomic abnormalities in HNSCC has limited the development of targeted therapies in the past [5]. Thus, identifying a reliable molecular biomarker to predict the prognosis of patients with HNSCC is an urgent task. To better treat HNSCC patients, many studies have focused on identifying relevant biomarkers to predict patient prognosis [6–8]. However, because the human body is a complex organism and the occurrence and development of cancer may involve many aspects, the limitations of mining disease-related factors based on the one-omics perspective have become increasingly apparent in recent years. Additionally, constantly improving databases provide technical support, enabling multiomics studies involving genomics, transcriptomics, and proteomics. Therefore, the application of multiomics data systems to explore cancer biomarkers has become an important trend in precision medicine, allowing joint research on macro and micro aspects.

The enhancer of polycomb homolog 1 (*EPC1*) has a protective function against DNA damage. Epigenetic factor *EPC1* is a master regulator of the DNA damage response by interacting with *E2F1* to silence cell death and activate metastasis-related gene signatures [9]. Pathways known to be associated with this gene include chromatin-modifying enzymes, chromatin organization, and histone acetyl transferases (HATs) [10]. Sophisticated studies have demonstrated that *EPC1* is involved in the NuA4 HAT complex, and the crystal structure and molecular basis for *EPC1* bound to MBTD1 were determined [11]. Additionally, hsa_circ_0007919 knockdown resulted in hsa-let-7a downregulating *EPC1* mRNA [12]. According to previous reports, abnormal *EPC1* was present in both endometrial stromal sarcoma [13, 14] and ossifying fibromyxoid tumors [15], whereas *EPC1* silencing inhibited lung cancer cell proliferation and tumor growth [16]. Additionally, *EPC1* has been correlated with patient prognosis in microarray screenings of nasopharyngeal cancer [17]. These data suggest that *EPC1* may be a prognostic biomarker that is worth studying. Therefore, we aimed to provide further insight into the prognostic significance of *EPC1* in patients with HNSCC and comprehensively analyze *EPC1* from a multiomics perspective to explore its mechanisms.

## 2. Materials and Methods

### 2.1. Patients and Transcriptional Expression Profile

The clinical data and gene expression profiles of patients with HNSCC were downloaded from the Genomic Data Commons. Clinical data were mainly used for survival analysis, and gene expression profiles were used for subsequent multiomics analyses. This study was conducted in accordance with TCGA publication guidelines.

### 2.2. The Associations between Gene Expression and Key Prognostic Factors

To analyze possible associations between clinical parameters and *EPC1* expression, the TISIDB database (http://cis.hku.hk/TISIDB/, last accessed on 31 July 2022), which integrated clinical data from TCGA, was used to identify differences in *EPC1* expression between different HNSCC subtypes [18]. Survival curves associated with *EPC1* expression in HNSCC were plotted using the Kaplan-Meier plotter (http://kmplot.com/analysis/, last accessed on 31 July 2022) [19]. Overall survival (OS) was selected for the HNSCC clinical endpoint analysis. OS was defined as the period from the date of diagnosis to the date of death from any cause [20]. UALCAN (http://ualcan.path.uab.edu/, last accessed on 31 July 2022) is a web platform based on JavaScript, CSS, and PERL-CGI. Differences in *EPC1* expression in HNSCC can be visualized using UALCAN according to TP53 mutation status or the presence of HPV [21]. TIMER 2.0 (http://timer.comp-genomics.org/, last accessed on 31 July 2022) was used to analyze three sample types: total HNSCC samples, HPV-positive HNSCC samples, and HPV-negative HNSCC samples. Therefore, the association between *EPC1* and clinical outcomes of patients with HNSCC was investigated based on the presence of HPV [22]. At the same time, the Wilcoxon rank sum test was used to analyze the relationship between the *EPC1* and TP53 mutation status.

### 2.3. Screening and Functional Enrichment of *EPC1* Expression-Related Genes

LinkedOmics (http://www.linkedomics.org/login.php, last accessed on 31 July 2022) includes multiomics data that were used to screen differentially expressed genes related to *EPC1* in HNSCC. Spearman's correlation test was used to predict*EPC1* association results. Volcano plots were visualized using the “chart-studio” package in Python 3.8.7. We then performed gene set enrichment analysis (GSEA 4.2.3). The rank criteria were a *P* value < 0.05, a false discovery rate (FDR) ≤ 0.05, a minimum number of genes (size) = 5, and simulations = 500. Gene Ontology (GO) and Kyoto Encyclopedia of Genes and Genomes (KEGG) pathway enrichment analyses were performed. This methodology was used to explore the mechanism of *EPC1* at the gene expression level [23].

### 2.4. Construction of a Competing Endogenous RNA (ceRNA) Network

To explore the interaction of *EPC1* with long noncoding RNAs (lncRNAs) and microRNAs (miRNAs), DIANA-tools and other databases were used to construct a ceRNA network. DIANA-tools contain TarBase v.8 and LncBase v.3 databases. TarBase v.8 (http://www.microrna.gr/tarbase, last accessed on 4 August 2022) is a database of miRNA-gene interactions confirmed by experiments [24]. TarBase v.8 and the CancerMIRNome database (http://bioinfo.jialab-ucr.org/CancerMIRNome/, last accessed on 5 August 2022) were used to screen *EPC1* gene-related miRNAs in the HNSCC samples [25]. Lnc2Cancer 3.0 (http://www.bio-bigdata.net/lnc2cancer/, last accessed on 5 August 2022) database was used to obtain experimentally validated HNSCC-related lncRNAs [26]. LncBase v.3 (https://diana.e-ce.uth.gr/lncbasev3, last accessed on 5 August 2022) was used as a tool to determine the relationship between miRNAs and lncRNAs [27]. Finally, a lncRNA-miRNA-mRNA Sankey diagram was plotted using SangerBox software (http://vip.sangerbox.com/, last accessed on 4 August 2022).

### 2.5. Protein-Protein Interaction (PPI) Networks and KEGG Pathway Enrichment

To reveal the role of *EPC1* in proteomics, *EPC1*-related proteins in HNSCC were screened using LinkedOmics (set at *P* < 0.05) and used to construct *EPC1*-related PPI networks using the STRING database [28] (https://string-db.org/, last accessed on 6 October 2020). In addition, KEGG pathway enrichment was used to predict the role of *EPC1* at the protein level in HNSCC, which was visualized using the “ggplot2” package of R software 3.6.3.

### 2.6. *EPC1*-Related Immune Cells and Immunoreactive Substances in HNSCC Samples

TIMER 2.0 was used to systematically analyze immune infiltration. The tool integrated CIBERSORT-ABS with published existing data [29]. The associations between immune infiltration and *EPC1* gene expression in HNSCC patients (HNSCC, HPV-positive HNSCC, and HPV-negative HNSCC) were explored.

### 2.7. Statistical Analysis

Kaplan-Meier curves were used to compare the differences in survival time. OS was selected for the HNSCC clinical endpoint analysis. Hazard ratios (HRs) and their corresponding 95% confidence intervals (CIs) were calculated to assess the role of the *EPC1*. A log-rank test (*P* < 0.05) indicated a significant survival time difference. The results were verified using another database [30]. We also performed GSEA. And the rank criteria were a *P* value <0.05, a minimum number of genes size=5, stimulations=500, and aFDR ≤ 0.05. Additionally, the spearman correlation analysis and the wilcoxon rank-sum test were applied to show the correlations between the *EPC1* gene and other factors.

## 3. Results

### 3.1. Effect of the Differential Expression of *EPC1* on the Prognosis and Clinical Outcomes of Patients with HNSCC

**Figure 1:**
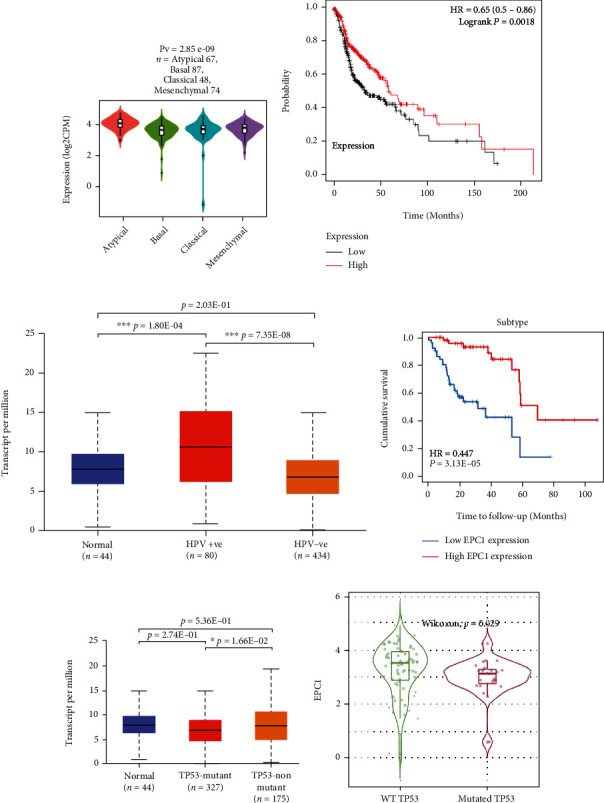
Correlations between the differential expression of the *EPC1* gene and key prognostic factors of HNSCC (TISIDB, Kaplan-Meier plotter, UALCAN, and TIMER 2.0 databases). Note: (a) expression of *EPC1* in different subtypes of head and neck tumors, (b) survival curve for *EPC1* expression levels, (c) differential expression of *EPC1* in head and neck tumors based on the presence of HPV, (d) survival curve for *EPC1* expression in HPV-positive head and neck cancer samples, (e) differential expression of *EPC1* in head and neck cancer based on TP53 mutation status, and (f) association between *EPC1* and TP53 mutations in HPV-positive head and neck cancer samples.

Using the TISIDB platform, spearman correlation analysis was performed to study the association between *EPC1* expression and HNSCC subtypes. *EPC1* expression levels were not equal among the different subtypes (Figure 1(a)). The Kaplan-Meier plotter platform was used to analyze the association between survival and *EPC1* expression (Figure 1(b)). The median survival time for the low-*EPC1* expression group was 33.10 months, and the median survival time for the high-*EPC1* expression group was 61.27 months; the difference was statistically significant (HR < 1, *P* < 0.01), suggesting that HNSCC patients with high *EPC1* expression have a better prognosis.

Based on TCGA samples and the UALCAN website, *EPC1* expression in HPV positive HNSCC tumors was not only significantly higher than that in paracancerous tissues (*P* < 0.01) but also significantly higher than that in HPV negative HNSCC samples (*P* < 0.01) (Figure 1(c)). Using HPV positive HNSCC samples, we explored the relationship between *EPC1* expression and patient prognosis. The results showed that patient prognosis was significantly better with higher *EPC1* expression (Figure 1(d)) (*P* < 0.01). However, no significant effect of *EPC1* expression on patient prognosis was found when HPV negative HNSCC samples were analyzed. In addition, compared with that in TP53-mutated HNSCC, *EPC1* expression in wild-type TP53 HNSCC was significantly higher (*P* < 0.05) (Figure 1(e)). The expression of wild-type TP53 *EPC1* was relatively high in HPV positive HNSCC samples. In HPV negative HNSCC samples, no significant difference in *EPC1* expression was identified between the wild-type TP53 and TP53-mutated samples (Figure 1(f)). Therefore, we hypothesized that high *EPC1* expression was suggestive of better prognosis in patients with HNSCC, because it tended to be present more often in HNSCC patients with HPV positive, who were more sensitive to radiotherapy and had a greater prognosis compared with HNSCC patients without HPV.

### 3.2. Screening and Functional Prediction of Genes Associated with the Differential Expression of *EPC1* in HNSCC

**Figure 2:**
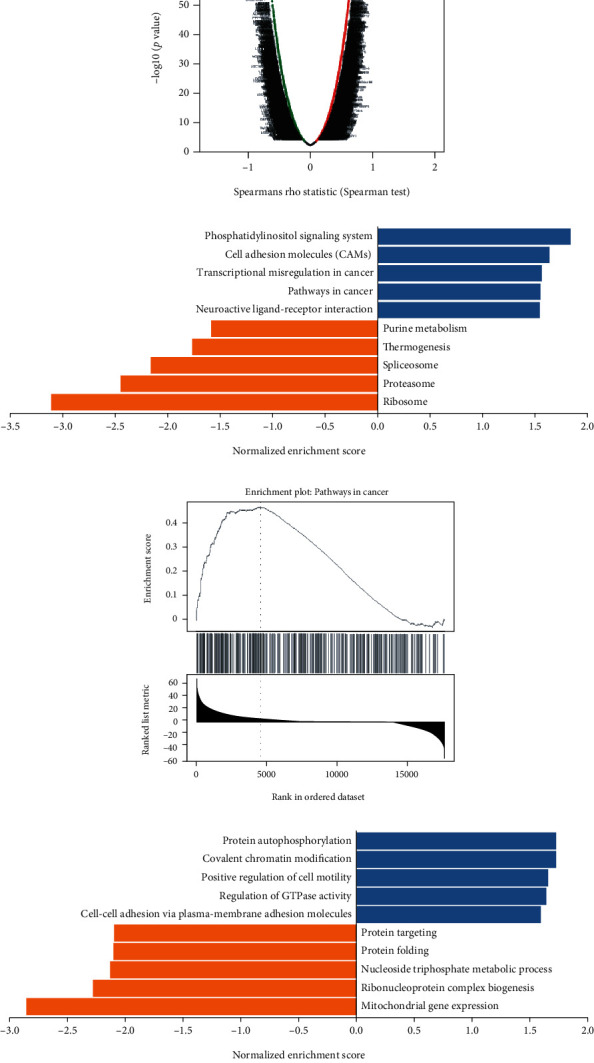
Screening and enrichment analysis of *EPC1-*related genes in HNSCC samples (LinkedOmics database). Note: (a) volcano plots for *EPC1*-related genes in head and neck cancer samples. (b) KEGG pathway enrichment analysis of *EPC1-*related genes in head and neck cancer samples. (c) Enrichment plots (GSEA) for pathways in cancer. (d) Enrichment of *EPC1*-related genes in biological processes.

LinkedOmics was used to screen genes that were significantly positively correlated with the *EPC1* and genes that were significantly negatively correlated with *EPC1*. A total of 20,164 related genes were obtained, including 8208 genes with negative correlations and 11,956 genes with positive correlations, and volcano plots were constructed (Figure 2(a)). Notable positively correlated genes included *ZNF41*, *NR2C2*, and *CEP350*. Notable negatively correlated genes included *MRPL28, C14orf156*, and *TMEM280*. After obtaining the gene dataset, we performed GSEA. The rank criteria were a *P* value < 0.05, FDR ≤ 0.05, minimum number of genes (size) = 5, and simulations = 500. KEGG pathway analysis was also performed. We selected “redundancy reduction: weighted set cover” and screened 5 positively correlated KEGG pathways (labeled blue, Figure 2(b)): phosphatidylinositol signaling system, cell adhesion molecules, transcriptional misregulation in cancer, pathways in cancer, and neuroactive ligand-receptor interaction. We also screened five negatively correlated KEGG pathways (labeled orange, Figure 2(b)): purine metabolism, thermogenesis, spliceosome, proteasome, and ribosome. Using pathways in cancer as an example, 190 genes were enriched (enrichment score = 0.48; normalized enrichment score = 1.55; *P* < 0.01), and the difference was statistically significant (Figure 2(c)). The above steps were repeated for the GO analysis (biological processes). Five positively correlated biological processes were screened (labelled blue, Figure 2(d)): protein autophosphorylation, covalent chromatin modification, positive regulation of cell motility, regulation of GTPase activity, and cell-cell adhesion via plasma-membrane adhesion molecules. Additionally, five negatively correlated biological processes were screened (labeled orange, Figure 2(d)): protein targeting, protein folding, nucleoside triphosphate metabolism, ribonucleoprotein complex biogenesis, and mitochondrial gene expression. The above analyses showed that the differential expression of *EPC1* at the gene level was related to cancer pathways. In addition, functional enrichment results showed that *EPC1* expression was mainly related to protein phosphorylation and cell adhesion.

### 3.3. Construction of a lncRNA-miRNA-mRNA Network Based on the Differential Expression of *EPC1* in HNSCC Samples

**Figure 3:**
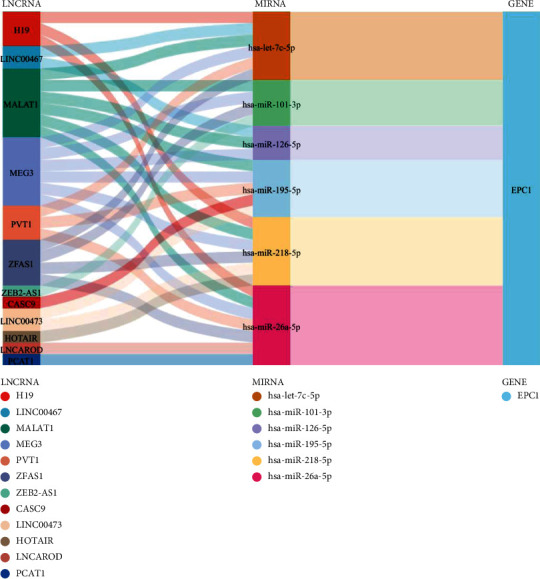
*EPC1*-related miRNAs and lncRNAs in HNSCC samples (CancerMIRNome, TarBase v.8, LncBase v.3, and Lnc2Cancer 3.0). Note: *EPC1* gene-related lncRNA-miRNA-mRNA network diagram.

49 *EPC1* gene-related miRNAs were identified using the TarBase V. 8 database, and the CancerMIRNome platform uncovered 168 miRNAs were differentially expressed in HNSCC. The two datasets intersected, resulting in 13 overlapping miRNAs. The Lnc2Cancer 3.0 database was used to obtain 43 experimentally verified HNSCC-related lncRNAs, and the LncBase v3 was applied to discover experimentally supported miRNA-lncRNA interactions. Taken together, 12 lncRNAs (H19, LINC00467, MALAT1, MEG3, PVT1, ZEB2-AS1, ZFAS1, LINC00473, HOTAIR, PCAT1, CASC9, and lncAROD) and 6 miRNAs (hsa-miR-26a-5p, hsa-let-7c-5p, hsa-miR-126-5p, hsa-miR-195-5p, hsa-miR-218-5p, and hsa-miR-101-3p) were screened (Figure 3).

### 3.4. Screening and Pathway Enrichment of Proteins Related to Differential *EPC1* Expression in HNSCC

**Figure 4:**
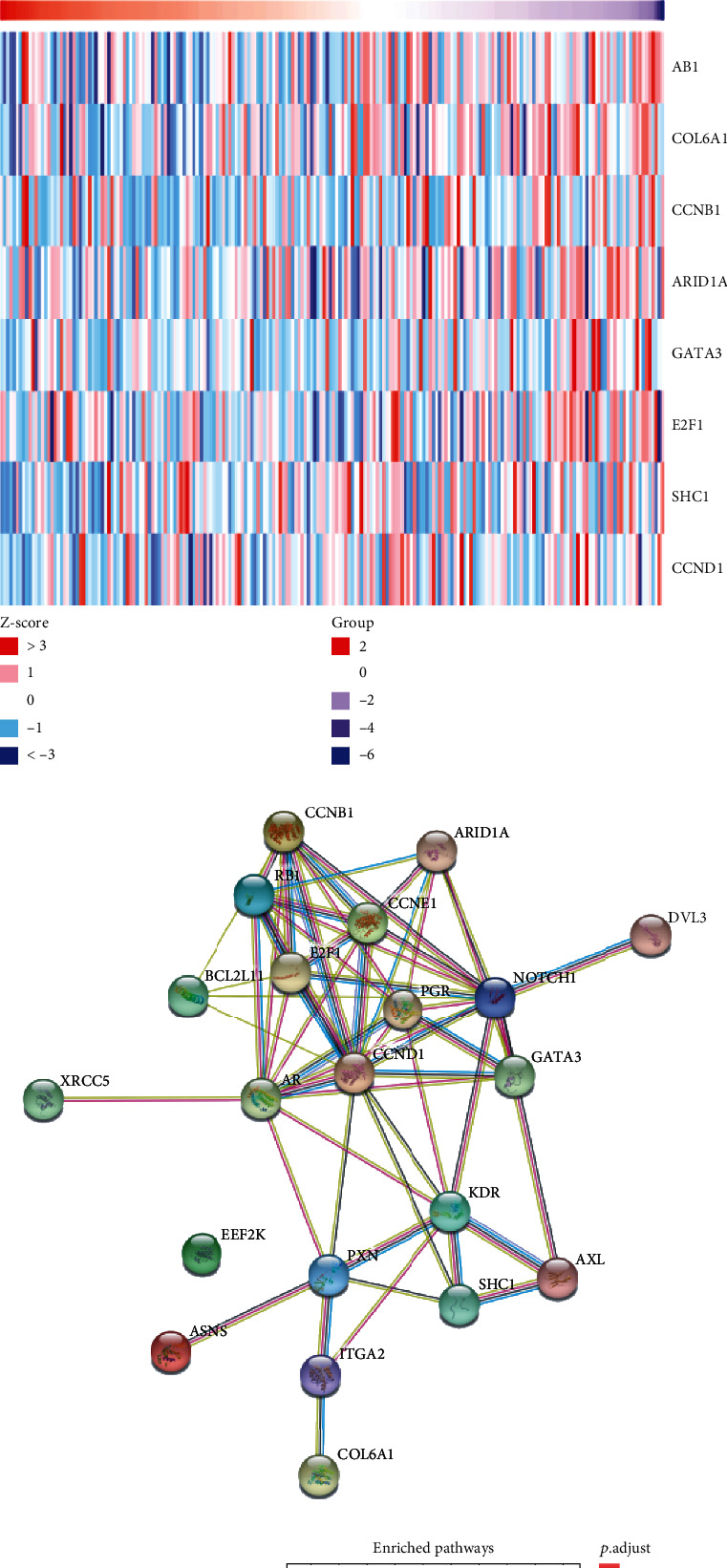
Proteomics study of *EPC1* in HNSCC samples (LinkedOmics and STRING databases). Note: (a) Proteins positively associated with *EPC1* expression in head and neck cancer samples, (b) proteins negatively correlated with *EPC1* expression in head and neck cancer samples, (c) protein-protein interaction network constructed from related proteins, and (d) bubble diagram of pathways enriched with related proteins.

LinkedOmics was used to screen 13 proteins that were positively related to *EPC1* gene expression (Figure 4(a)) and 7 proteins that were negatively related to *EPC1* expression (Figure 4(b)), all of which met the criteria of*P* < 0.05. The corresponding heat maps were drawn. Using the STRING database, an interaction network consisting of 21 proteins was constructed (Figure 4(c)) and protein enrichment analysis was used to obtain the top 10 related pathways based on gene ratios (Figure 4(d)). The PPI network suggested that proteins coexpressed with *EPC1* might be involved in various cancer-related signaling pathways, such as HPV infection, endocrine resistance, cell cycle disruption, plaque adhesion, breast cancer, gastric cancer, hepatocellular carcinoma, pancreatic cancer, and small-cell lung cancer.

### 3.5. Differential Expression of *EPC1* among All HNSCC Samples, HPV-Positive HNSCC Samples, and HPV-Negative HNSCC Samples and the Association with Immunity

At the cellular level, HNSCC, HPV positive HNSCC, and HPV negative HNSCC samples were subjected to immunological analysis using the TIMER 2.0 platform. We used CIBERSORT-ABS algorithm for immune scoring analysis. After adjusting for tumor purity, 6 *kinds of EPC1*-related immune cells were screened out from the HNSCC samples under the restriction condition that one of three sample types reached |rho| > 0.3 and *P* < 0.05. Except for alternatively activated macrophages (M2), we found *EPC1* gene expression in HNSCC patients with HPV infection showed a higher infiltration levels of most immune cell (Table 1).

**Figure 5:**
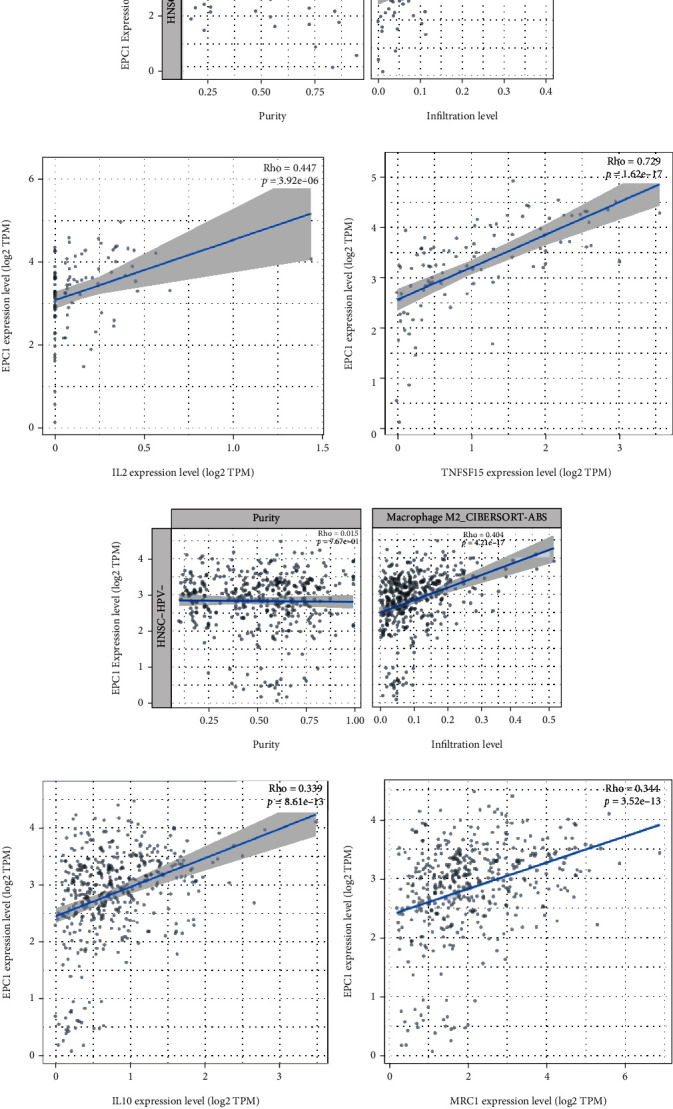
*EPC1*-related immunoreactive substances (HNSCC samples; TIMER 2.0). Note: (a) *EPC1* was positively correlated with *M1* (HPV-positive HNSCC samples). (b) *EPC1* was positively correlated with IL2 (HPV-positive HNSCC samples). (c) *EPC1* was positively correlated with *TNFSF15* (HPV-positive HNSCC samples). (d) *EPC1* was positively correlated with *M2* (HPV-negative HNSCC samples). (e) *EPC1* was positively correlated with IL10 (HPV-negative HNSCC samples). (f) *EPC1* was positively correlated with MRC1 (HPV-negative HNSCC samples).

To further explore the correlation between macrophages and *EPC1*, we focused on classically activated macrophage (M1), M2, and their related molecules. Among HPV positive HNSCC patients, *EPC1* gene expression was positively associated with the infiltration level of M1 (Figure 5(a)). Also, *EPC1* gene expression level was correlated with IL2 (Figure 5(b)) and TNFSF15 (Figure 5(c)). However, M2 macrophage infiltration level was positively associated with *EPC1* gene in HNSCC patients without HPV infection (Figure 5(d)), compared with HNSCC patients with HPV infection. And, *EPC1* gene expression in HPV HNSCC patients was associated with IL10 (Figure 5(e)) and MRC1 (Figure 5(f)). These results suggested that *EPC1* may alter the tumor microenvironment of HNSCC by affecting the immune cells and immune-related molecules.

## 4. Discussion

*EPC1* is a multicomb homolog 1 (*Drosophila*) enhancer involved in the regulation of cell growth and transcription [31]. *EPC1* anomalies may be involved in ossifying fibromyxoid tumors [15], endometrial stromal sarcoma [32], pancreatic cancer [33], and nasopharyngeal carcinoma (NPC) [34]. However, the effect of this gene on the survival prognosis of HNSCC patients has not been studied. Therefore, we tried to identify the function of *EPC1* as a prognostic biomarker in HNSCC by multiomics integrative analysis. We found that *EPC1* might indicate a better overall survival for patients with HNSCC, which was inconsistent with the results of NPC from our study [34]. We considered that one of the reasons might be that the expression data of NPC was not available in TCGA, due to the rarity of NPC in America. In addition, we found that high *EPC1* expression was correlated with HPV-positive and non-tp53 mutations, both of which were favorable prognostic factors in HNSCC [35]. In addition, coexpressed genes, competing endogenous RNAs, protein interaction networks, immune cells, and molecules may also participate in the prognosis of HNSCC patients.

The survival time of HNSCC patients with high *EPC1* expression is longer, which was not noted in previous studies. *EPC1* expression varied in different HNSCC subtypes. And *EPC1* expression was relatively high in HPV positive HNSCC samples. In addition, among the *EPC1*-related protein enrichment pathways, HPV infection-related pathways predominated, suggesting that *EPC1* and HPV may have a certain correlation that affects patient prognosis. In addition, TP53 mutations often indicate poor prognosis, and *EPC1* expression is relatively low in HNSCC with TP53 mutations. However, *EPC1* expression was relatively high in wild-type TP53 samples, suggesting that high *EPC1* expression may indicate the better prognosis of patients with HNSCC.

To further investigate the possible mechanism by which *EPC1* affects HNSCC prognosis, we performed a multiomics analysis of *EPC1*. At the gene level, *EPC1*-related gene enrichment results indicated that *EPC1* participates in some important biological processes, such as protein phosphorylation, cancer-related pathways, and cell mobility. A study revealed that oxidative phosphorylation was significantly enriched in HPV^+^ HNSCC, which was one of the differences from HPV^−^ HNSCC [36]. Therefore, we can speculate that HPV may be an important reason for the close relationship between *EPC1* gene expression and protein autophosphorylation. In transcriptomics studies, lncRNA H19 was an important link in the *EPC1*-related lncRNA-miRNA-mRNA network. High lncRNA H19 expression was positively correlated with the growth, migration, and invasion of lung tumor cells, but low H19 expression is associated with poor prognosis for patients with microinvasive follicular thyroid carcinoma and can be used to predict distant metastasis [37]. These results suggest that *EPC1* may affect the prognosis of patients with HNSCC through lncRNA H19, leading to different prognoses in patients with different cancers. Besides, H19 [38] and MALAT1 [39] were upregulated in HPV^+^ cancers than those in HPV^−^ cancers, which indicated that HPV might participate in the regulation of the ceRNA network. Proteomics studies have shown that *EPC1*-related proteins were mainly involved in HPV infection, endocrine resistance, cell cycle, and cancer pathways. In this study, high *EPC1* expression in HPV positive HNSCC samples had a significant positive effect on prognosis, suggesting that *EPC1* and HPV may be associated with patient survival prognosis. At the immunomics level, intratumoral immune status was a key factor affecting patient survival and response to immunotherapy. The tumor microenvironment has clinical significance in predicting therapeutic effects [40]. *EPC1*-related immune cells, such as macrophage, B cells, and T cells, may play a role in controlling tumor growth. Furthermore, M1 macrophage was considered to have greater antitumor activity [41], which was positively correlated with *EPC1* expression in HPV^+^ tumors. *IL2* served as an M1 marker and *TNFSF15* was shown to promote M1 production, both of which showed a correlation with *EPC1* expression. Besides, *EPC1* expression was more strongly correlated with M2 and its markers in HPV^−^ tumors than in HPV^+^ tumors. M2 was regarded as a tumor promoting factor in previous studies [42, 43]. Taken together, it may partly reveal why high *EPC1* expression indicates a better prognosis in HNSCC, especially in HPV^+^ tumors.

## 5. Conclusions

This study did not investigate the role of *EPC1* alone but rather investigated the differentially expressed genes, ceRNA networks, interacting proteins, and immune infiltration levels associated with *EPC1* in HNSCC samples. The data used in this study were experimentally validated or genetically sequenced, based on real-world data. Using these databases, we further confirmed that high *EPC1* expression was a favorable factor for the prognosis of patients with HNSCC. *EPC1* expression correlated with HPV infection, protein phosphorylation, and immune cell infiltration. These factors may explain the role of *EPC1* for HNSCC from multiple perspectives.

This study had some limitations. Data were obtained from public databases. However, the results have not yet been validated by animal model. In addiction, the functional role of *EPC1* in HNSC should be validated by overexpression and knockdown experiments in the future.

## Figures and Tables

**Table 1:** Correlation between *EPC1* expression and the level of immune infiltration.

Cell type	HNSCC (*n* = 522)	HNSCC-HPV^−^ (*n* = 422)	HNSCC-HPV^+^ (*n* = 98)
B cell naive	0.30	0.26	0.36
B cell plasma	0.28	0.23	0.39
Macrophage M1	0.35	0.28	0.53
Macrophage M2	0.40	0.40	0.32
T cell CD4^+^ memory resting	0.51	0.47	0.68
T cell CD8^+^	0.29	0.22	0.32

## Data Availability

The datasets used for the current study are available upon reasonable request from the corresponding authors.
